# Two negative experimental results and analysis of Alfvén’s theorem

**DOI:** 10.1371/journal.pone.0278990

**Published:** 2022-12-13

**Authors:** Zhu-Xing Liang, Yi Liang

**Affiliations:** 1 18-4-102 Shuixiehuadu, Zhufengdajie, Shijiazhuang, Hebei, China; 2 55-1-302 Shuixiehuadu, Zhufengdajie, Shijiazhuang, Hebei, China; Tongji University, CHINA

## Abstract

Alfvén’s theorem concerning “frozen-in magnetic field” states that the magnetic field lines, passing through an ideal conductive fluid, are frozen with the fluid, and the fluid can move along the magnetic field lines but cannot cross them. Here, we present two negative experimental results and an analysis of the theorem. The experimental results prove that Alfvén’s theorem cannot correctly explain the evolution of the magnetic field and magnetic resistance; therefore, it is incorrect. Mathematical analysis explains why this theorem is incorrect.

## Introduction

Alfvén’s theorem [1] is well known in the field of plasma physics and astrophysics. This theorem concerns the “frozen-in magnetic field” (hereafter, the frozen-in theorem) and describes two important phenomena regarding the behavior of magnetic field in an ideal conductive fluid: first, the magnetic flux is conserved in an ideal conductive fluid; and second, the magnetic field lines through an ideal conductive fluid are frozen within the fluid that can move along the magnetic field lines but cannot cross them.

After this theorem was put forward, it was widely recognized and appeared in almost all plasma physics textbooks [2–5].

Although this theorem was proposed by Alfvén, he advised against the use of his own theorem twice in his later papers [6, 7] and pointed out that it is often misleading. However, he did not explain why this was misleading.

Alfvén’s dissuasion did not reduce people’s trust in this theorem, and it is still widely used in plasma physics and astrophysics. For instance, a basic assumption of pulsar electrodynamics is that magnetosphere particles and magnetic field lines are frozen together [8].

Some new studies have carried out in-depth investigations of the frozen-in theorem, for instance, the test of the frozen-in theorem [9] and stochastic flux freezing [10, 11]. However, they have not demonstrated if it is misleading and why it is misleading.

We published a work on the frozen-in theorem [12] (hereafter LL2015) and presented our analysis and experimental results to demonstrate the problem with frozen-in theorem. Now, we add two new experimental results and a deeper analysis to further support the viewpoint in LL2015. The analysis of LL2015 mainly focused on the influence of polarized charges. The present analysis focuses on the influence of the system inductance.

Our aim is to suggest people to follow Alfvén’s advice and abandon Alfvén’s frozen-in theorem. This theorem has an important impact on the pulsar study. Once this theorem is abandoned, pulsar electrodynamics will need to be reconsidered.

## Experiment for the evolution of the magnetic field outside a conductor

**Fig 1:**
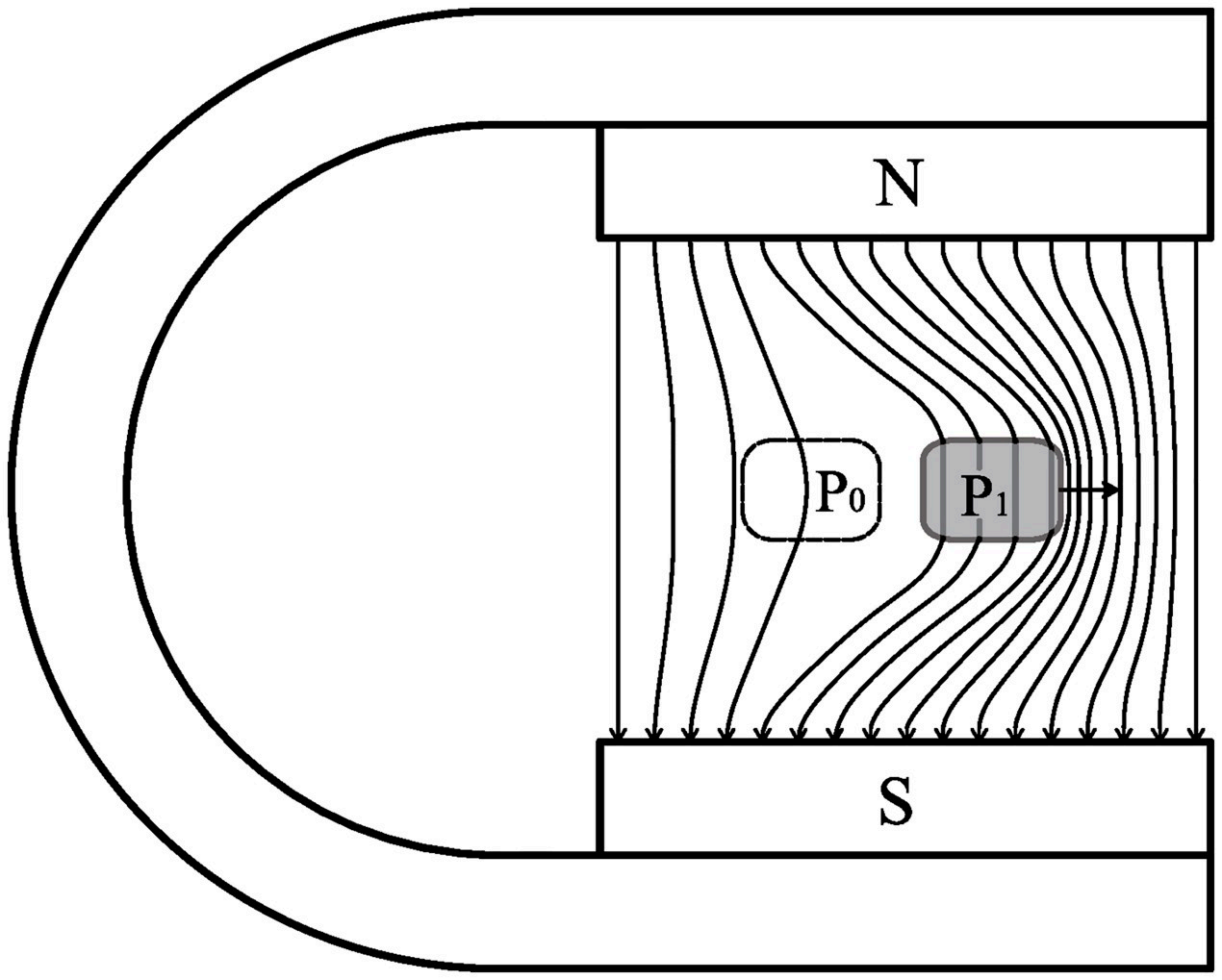
Evolution of the magnetic field lines expected by the frozen-in theorem. When an ideal conductor moves from *P*_0_ to *P*_1_, the front magnetic field lines are compressed while at the rear they spread apart.

If the frozen-in theorem holds, the phenomenon shown in Fig 1 will inevitably occur. When the ideal conductive fluid moves from point *P*_0_ to *P*_1_, the front magnetic field lines will become denser, and the rear magnetic field lines will become sparser. Our first experiment was to test whether this evolution of magnetic field lines occurred.

**Fig 2:**
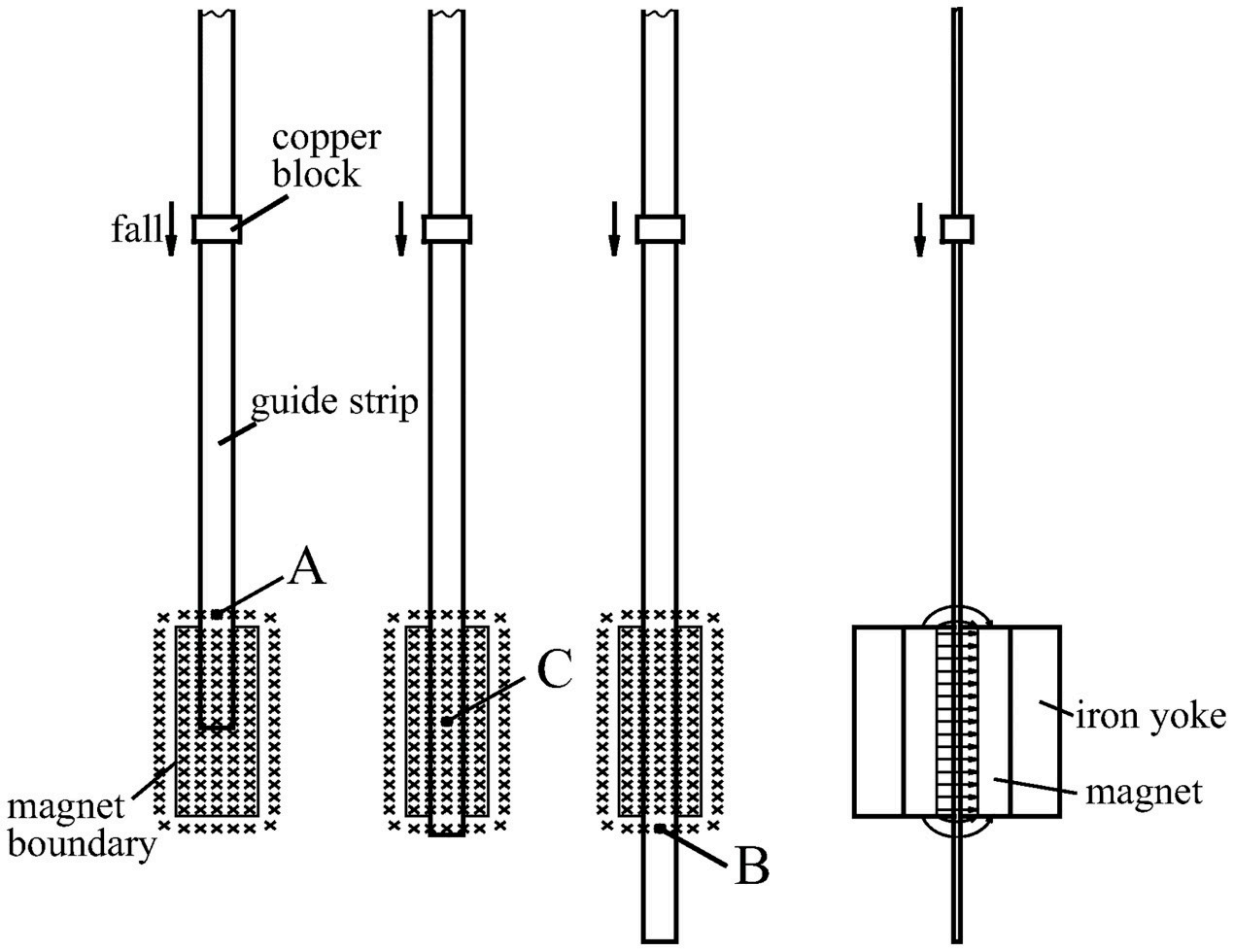
Schematic diagram for the evolution of magnetic field lines.

**Fig 3:**
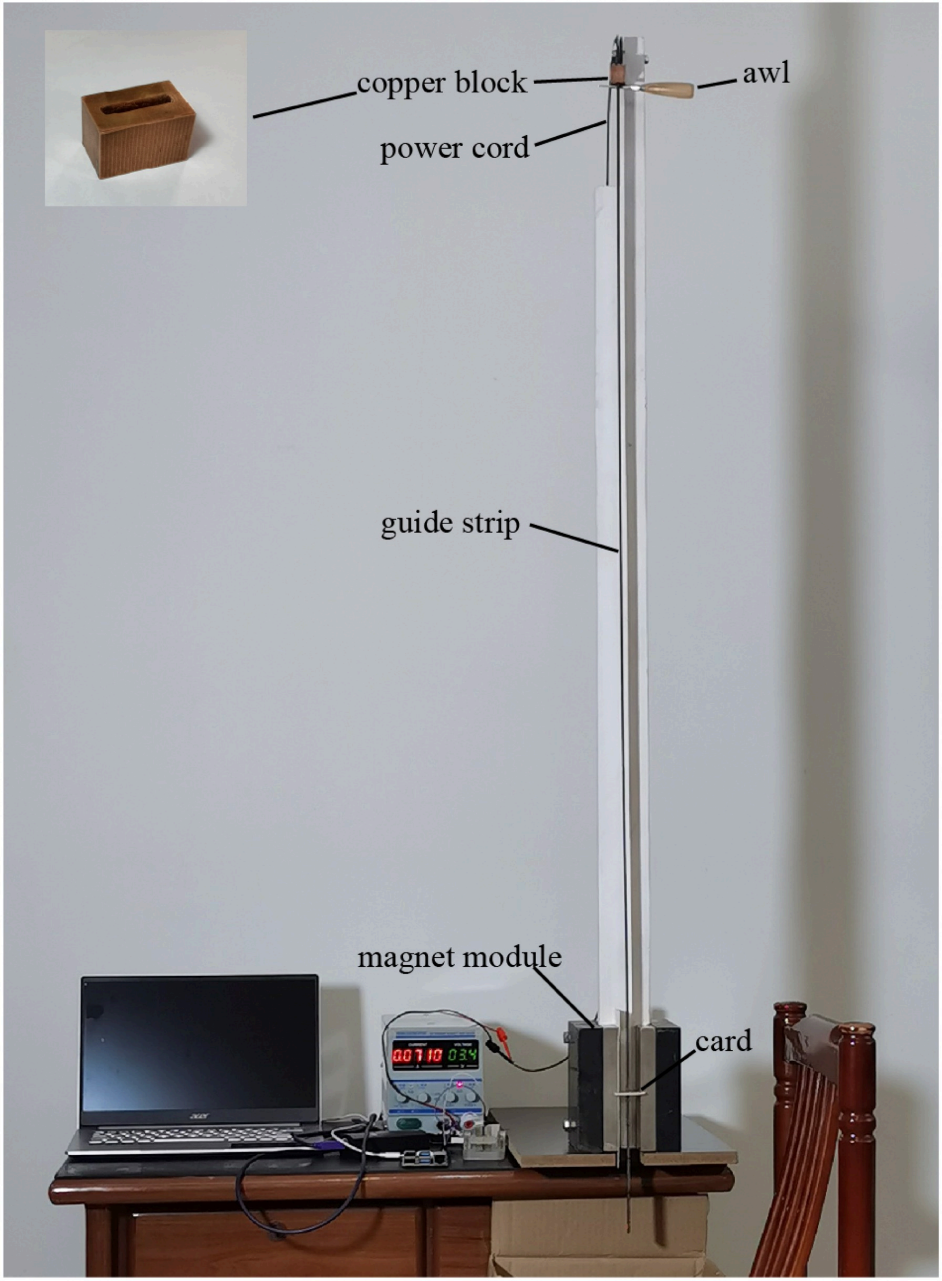
Optical image of the experiment device. When the awl is removed, the copper block falls along the guide strip and knocks the card out. The card is used to keep the guide strip in the central plane of the magnetic gap.

The device used in this experiment (Figs 2 and 3) included a rectangular copper block of size 28 × 18 × 20 mm, which was used instead of conductive fluid. Although the copper block differs from an ideal conductive fluid, it should lead to a similar trend in magnetic field line evolution. The frozen-in theorem was obtained by extrapolating from a solid conductor to a conductive fluid [13]. Therefore, in the following discussion, the conductor mentioned is also applicable to conductive fluids.

A rectangular hole in the center of the copper block (Fig 3) allowed it to fall approximately 1.2 m along the guide strip. The strip is composed of two layers of printed circuit boards and is soldered together using solder pads.

**Fig 4:**
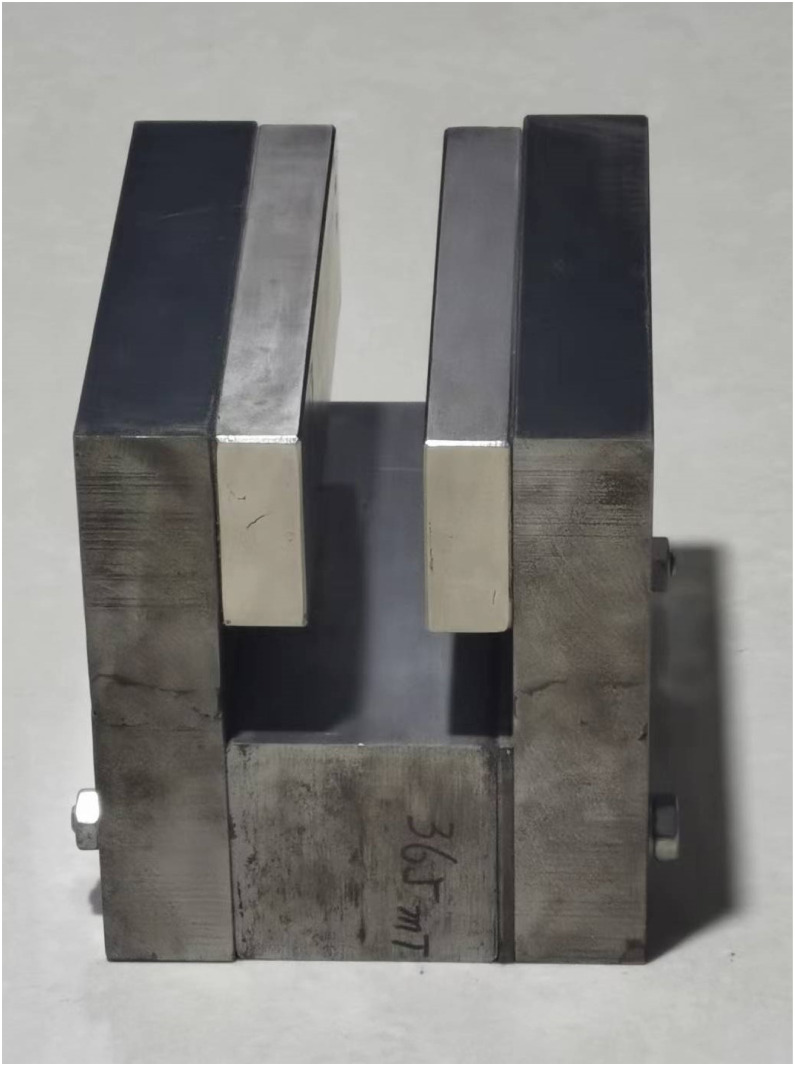
Magnetic module. The sizes of the strip-type magnets are 150 × 50 × 20 mm. The magnetic gap between the two magnets is 26 mm.

The magnetic module is composed of two strip-type magnets and an iron yoke (Fig 4). The website for purchasing magnets is: https://buyertrade.taobao.com/trade/detail/tradeSnap.htm?spm=a1z09.2.0.0.76002e8ddKmFoe&tradeID=551395245796065804&snapShot=true. The magnetic intensity at the center is approximately 0.36 T. Magnetic leakage around the magnet creates a gradient magnetic field; the field intensities at points A and B (Fig 2) are approximately 0.11 T. The distance from the edge of the magnet to point A or B was 10 mm.

**Fig 5:**
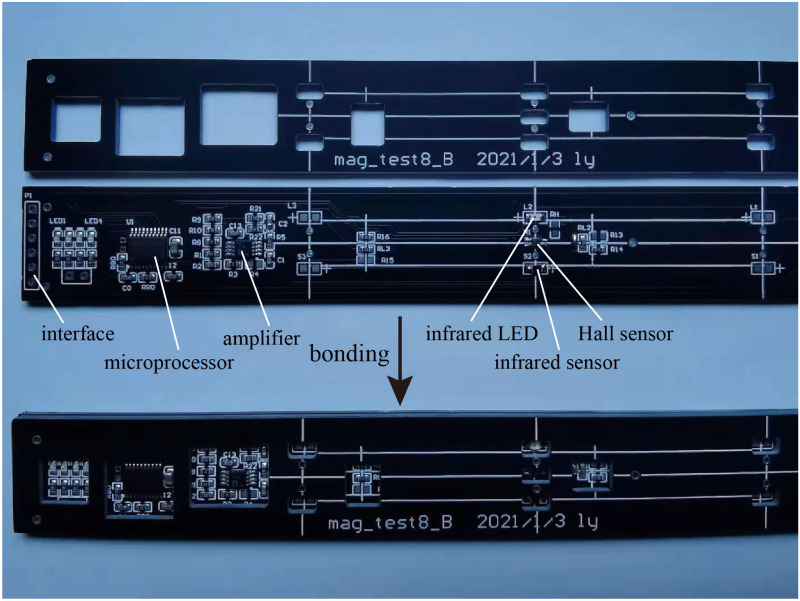
Photograph of the detection circuit.

The detection circuit (Fig 5) was located in the lower section of the guide strip. A Hall sensor was used to detect changes in the magnetic field, and the signal was recorded by the microprocessor. By changing the placement height of the guide strip, the Hall sensor can be placed at the detection points A, B, or C (Fig 2). The changes in the magnetic field were recorded when the copper block passed the Hall sensor. The fluctuation in the amplitude of the magnetic field outside the copper block is much lower than that of the static magnetic field. Therefore, between the Hall sensor and amplifier, AC coupling (with a time constant of 2 seconds) was used to block the DC component. A power supply was introduced from the top of the guide strip.

An infrared LED and an infrared sensor were placed near the Hall sensor. The light from the LED reached the sensor via an external reflective plate. When the copper block passed through the light field, a negative pulse signal was generated, which helped determine the relative position of the copper block and Hall sensor. The data generated by the Hall and infrared sensors were temporarily stored in the microprocessor and could be downloaded through the interface at the bottom of the detection circuit (Fig 5).

**Fig 6:**
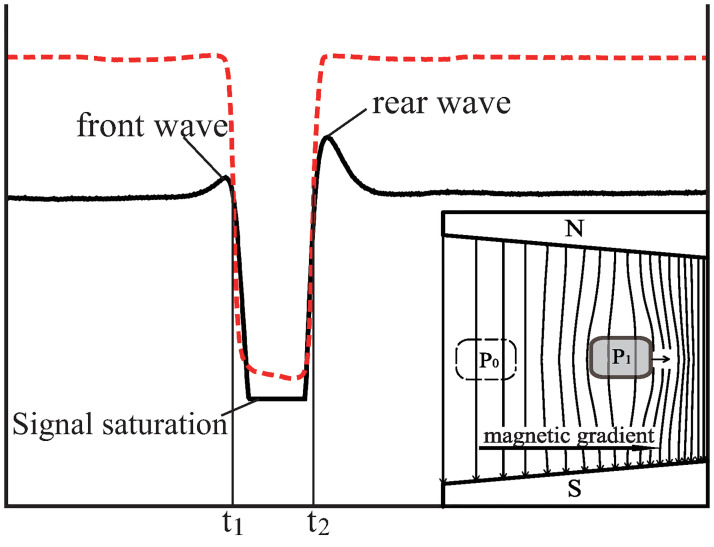
Magnetic field signal at the detection point A in Fig 2. The solid line is the magnetic signal, and the red dashed line is obtained from the infrared sensor. The inset illustrates the evolution of the magnetic field lines. The front and rear waves are both increasing waves, which means that the magnetic field lines in front of and behind the point *P*_1_ become denser.

**Fig 7:**
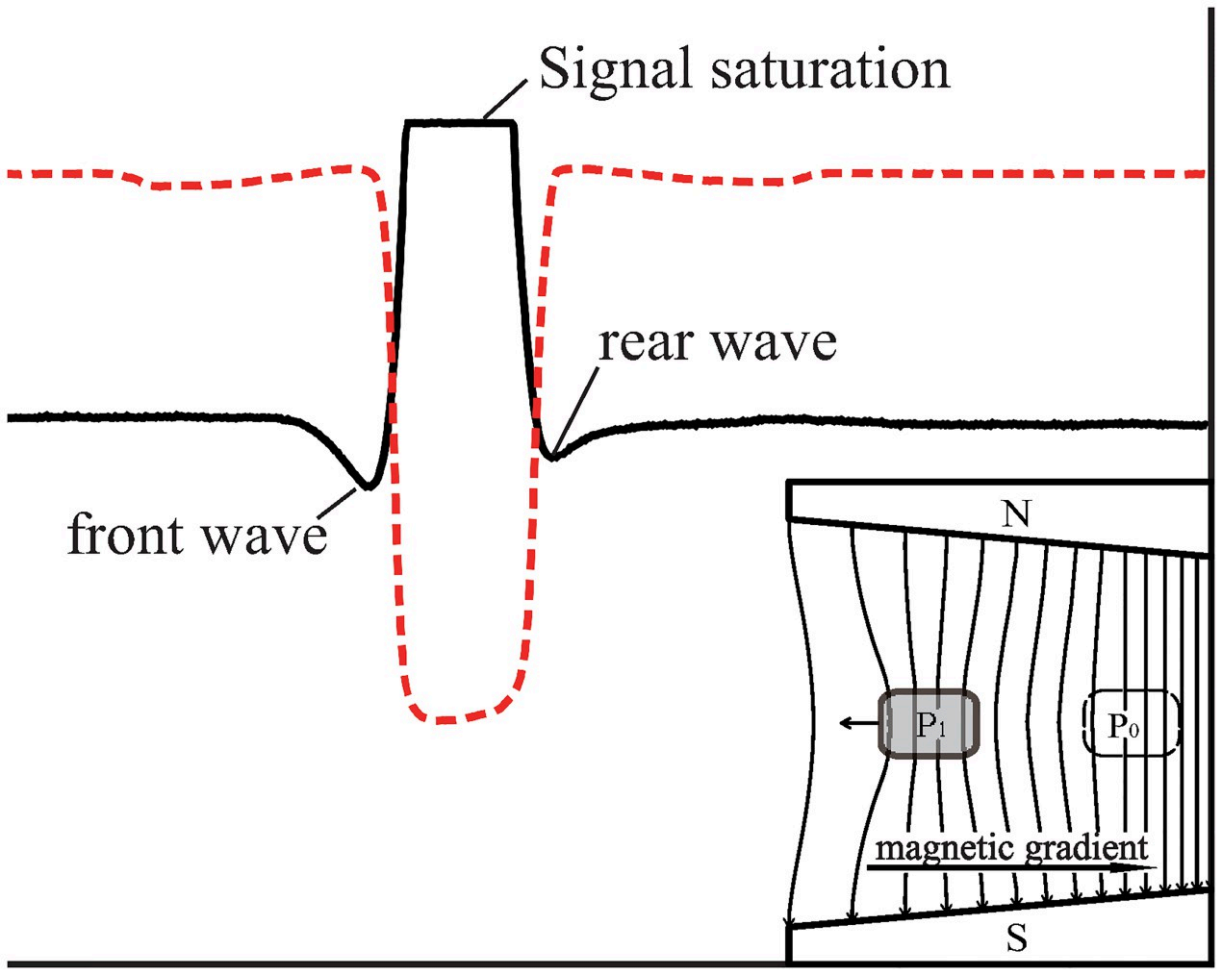
Magnetic field signal at point B. The front and rear waves are both in negative directions, indicating that not only do the magnetic field lines behind the copper block become sparse but also the ones in front become sparse, as shown in the inset.

**Fig 8:**
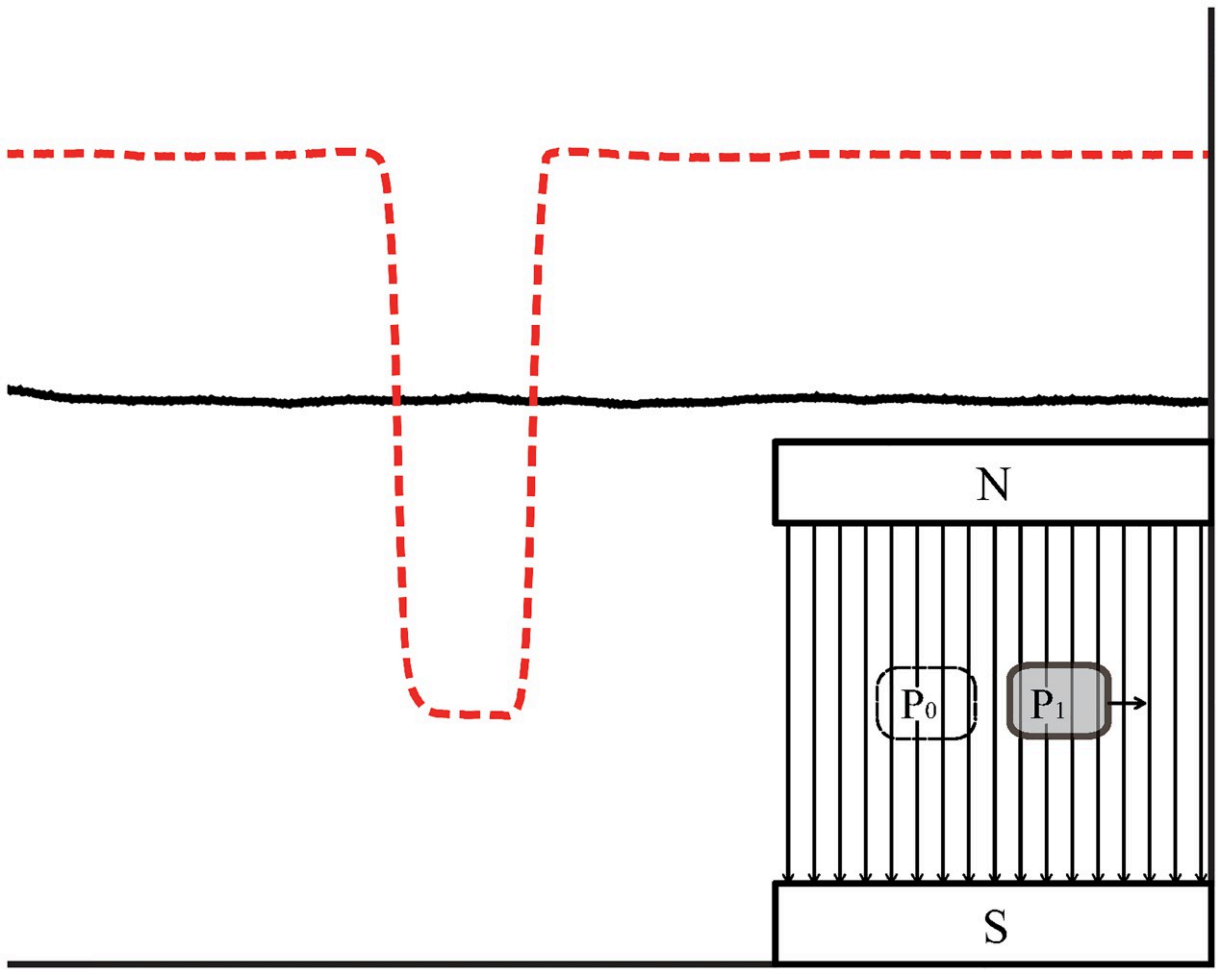
Magnetic field signal at point C. The black solid line is the magnetic field signal and has no obvious change. The red dashed line plots the signal of the infrared sensor.

The experimental results are plotted in Figs 6–8.

Initially, the Hall sensor was placed at point A, as shown in Fig 2. Fig 6 shows the signal recorded when the copper block fell. When the copper block had not reached point A before *t*_1_, the compression of the field lines in front of the copper block increased the density of the magnetic field lines around point A, thereby forming an increasing front wave.

From *t*_1_ to *t*_2_, the Hall sensor was inside the copper block and got a downward saturation signal, because the eddy current in the copper block was trying to maintain the original weak internal magnetic field.

After *t*_2_, the copper block passes through point A. The rear wave shows the magnetic-field variation at the rear of the copper block. If the frozen-in theorem holds, the magnetic field lines behind the copper block should become sparser (Fig 1) and the rear wave should be negatively directed instead of being positive (Fig 6). Hence, this result negates the frozen-in theorem.

From the signal of point B (Fig 7), the pushing effect expected by the frozen-in theorem did not appear. The magnetic field lines at the front of the copper block became sparser instead of getting denser (inset, Fig 7). Evidently, the frozen-in theorem is not supported.

The evolution of the magnetic field line at point C (Fig 8) shows that the magnetic field outside the copper block did not change when the copper block crossed a uniform magnetic field. This result again contradicts the frozen-in theorem.

From the above results, we find an obvious pattern:

1. If the copper block moves in the direction of the magnetic field gradient, the magnetic field lines at both the front and rear of the copper block become denser, as shown in the inset of Fig 6.
2. If the copper block moves against the magnetic gradient, the magnetic field lines at both the front and rear become sparser, as shown in the inset of Fig 7.
3. If the copper block moves along a path where the magnetic gradient is zero, all magnetic field lines outside the copper block remain unchanged, as shown in the inset of Fig 8.

Because both the front and rear magnetic changes are caused by the same eddy currents, they must simultaneously increase or decrease. The pattern expected from the frozen-in theorem (the front magnetic field lines become denser, and the rear magnetic field lines become sparser, as shown in Fig 1) will never appear.

This experiment proves that the argument shown in Fig 6 in LL2015 is correct.

## Magnetic damping experiment

According to the frozen-in theorem, a conductive fluid moving perpendicular to a magnetic field line is subjected to magnetic damping (drag resistance). The stronger the magnetic field, the greater is the magnetic damping. Nevertheless, LL2015 claims that a magnetic gradient (in the direction of motion) is a necessary condition for magnetic damping, and if no magnetic gradient, there will be no magnetic damping.

**Fig 9:**
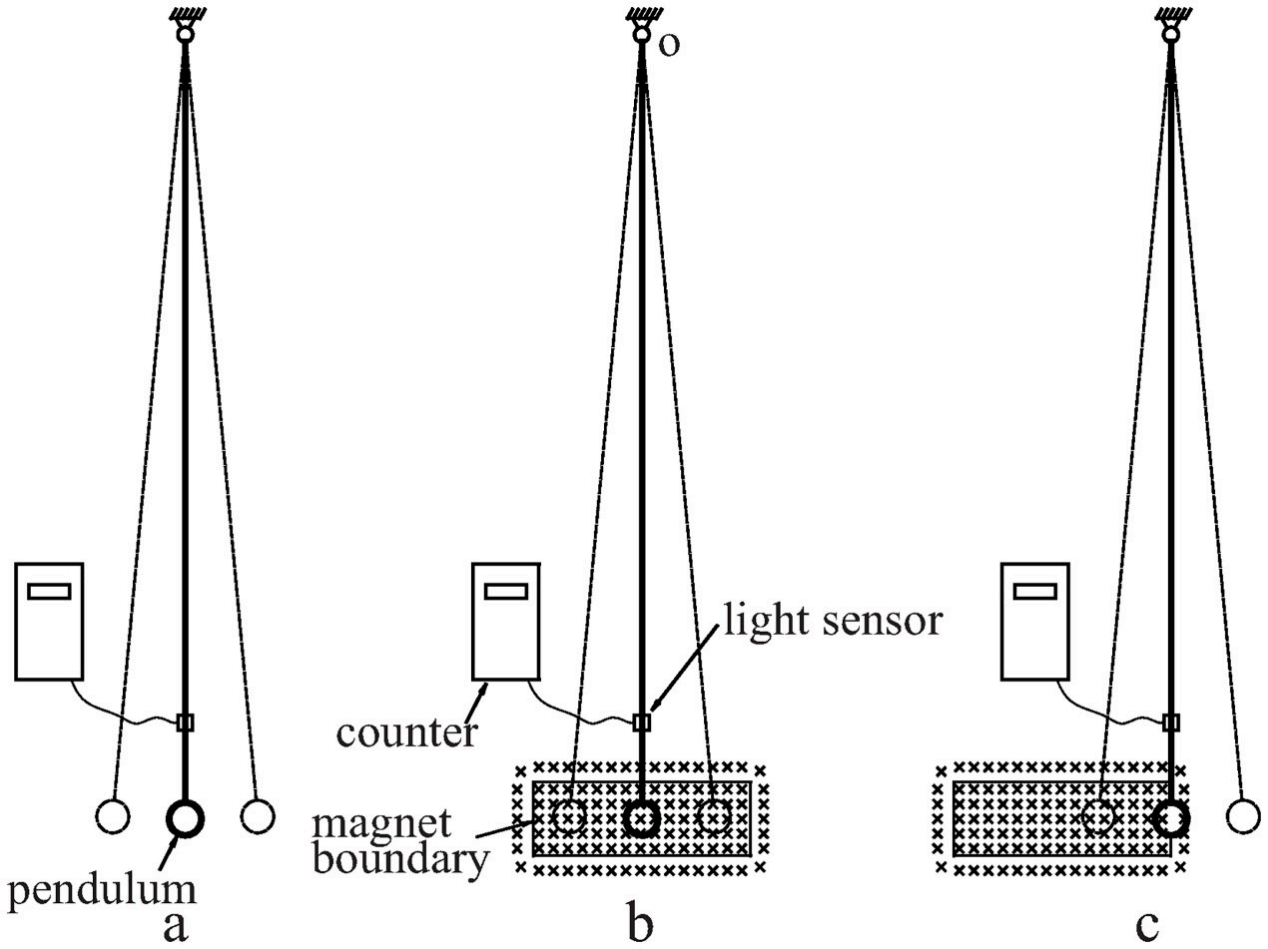
Schematics of the setup for the three damping experiments.

**Fig 10:**
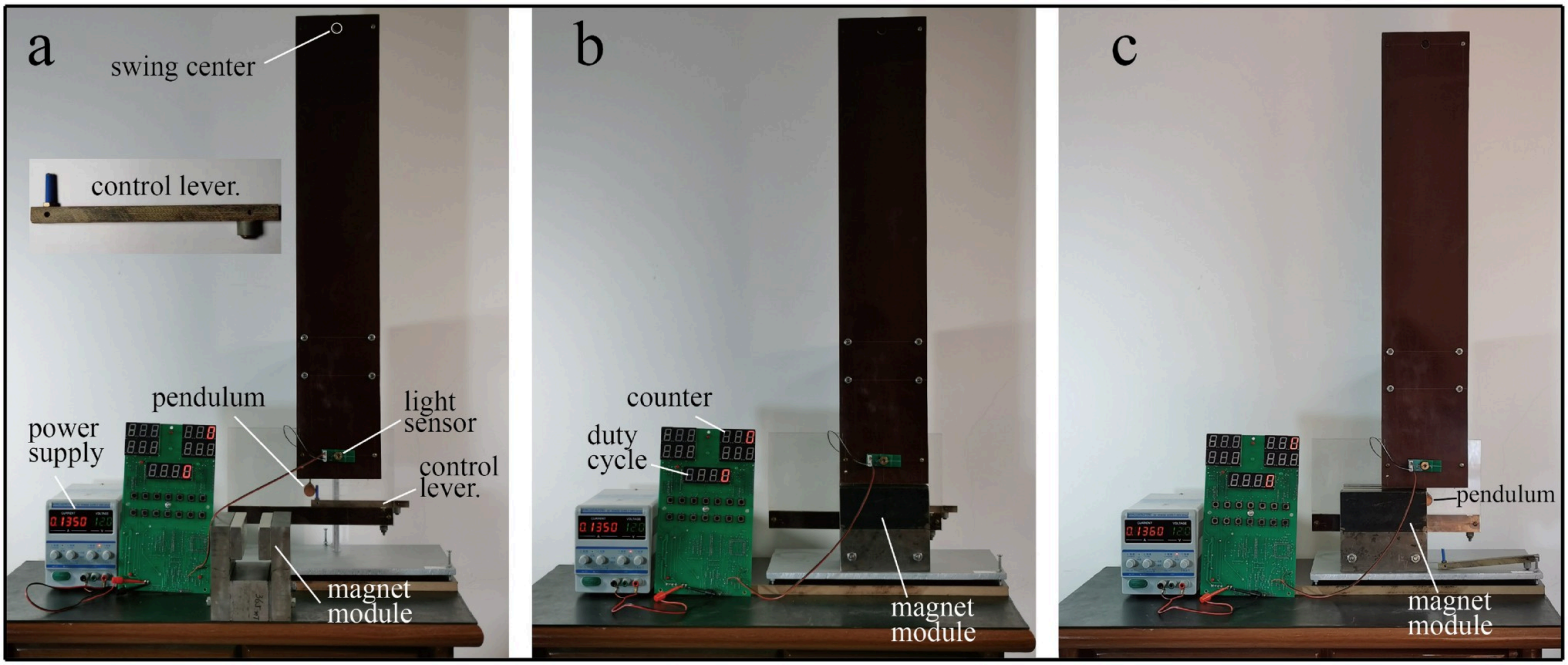
Optical images of the damping experimental device corresponding to the three experiments shown in Fig 9. As soon as the control lever is pulled out to the right, the pendulum starts swinging.

To verify which viewpoint is correct, we performed a series of pendulum tests (Figs 9 and 10). A copper pendulum with a copper rod swung around its fixed-point O. The length of the rod was approximately 80 cm. The tests covered three situations (a, b, and c in Fig 9). The pendulum oscillates in a) zero magnetic field, b) uniform magnetic field, and c) magnetic field with a gradient. In all three situations, the same pendulum and magnetic module (Fig 4) were used.

A control lever ensured that the starting angle of the pendulum was the same for each experiment. When the control lever was released, the pendulum started swinging. Subsequent friction and magnetic damping gradually reduced the swing amplitude. When the swing amplitude dropped to a certain value, the counting of swings stopped automatically. Because the frictional resistance was the same for all three situations, the swing count was negatively correlated with magnetic damping. The larger the magnetic damping, the smaller was the count.

The swinging rod blocks the light rays of the light sensor (Fig 10), thereby producing a positive pulse that is sent to the counter. The duty cycle of the pulse signal was related to the swing amplitude. The smaller the swing amplitude, the larger was the duty cycle. When the duty cycle was increased to 50%, the counter stopped automatically.

A self-made special counter was used for counting purpose. This counter needs a special function; that is, it can automatically stop counting according to the predetermined duty cycle. There was no suitable commodity in the market; therefore, we made it ourselves.

Ten runs were performed for each condition. Counts, mean values, and standard deviations (*σ*) are presented in Table 1.

**Table 1:** Counts of the pendulum’s swing.

Situation	Counts										Mean	*σ*
Zero magnetic field (a)	947	990	985	964	933	948	938	956	967	957	959	19
Uniform magnetic field (b)	843	838	850	842	849	840	827	852	846	851	844	7.6
Gradient magnetic field (c)	0	0	0	0	0	0	0	0	0	0	0	0

In the counts of the three situations, there is only a small difference between the uniform magnetic field and zero magnetic field. However, if the magnetic field has a gradient (situation c), a very strong damping occurs, and the swing stops within one cycle.

The small difference between situations a and b is because the magnet can only generate a quasi-uniform magnetic field instead of an ideal uniform magnetic field.

This pendulum experiment proves that the magnetic field gradient is a necessary condition for magnetic damping; when a conductor crosses a uniform magnetic field, it does not disturb the magnetic field, and the magnetic field does not impose magnetic resistance on the conductor.

## Discussions

The presented experimental results prove that the frozen-in theorem is incorrect. However, the question of why it is incorrect requires further theoretical analysis.

**Fig 11:**
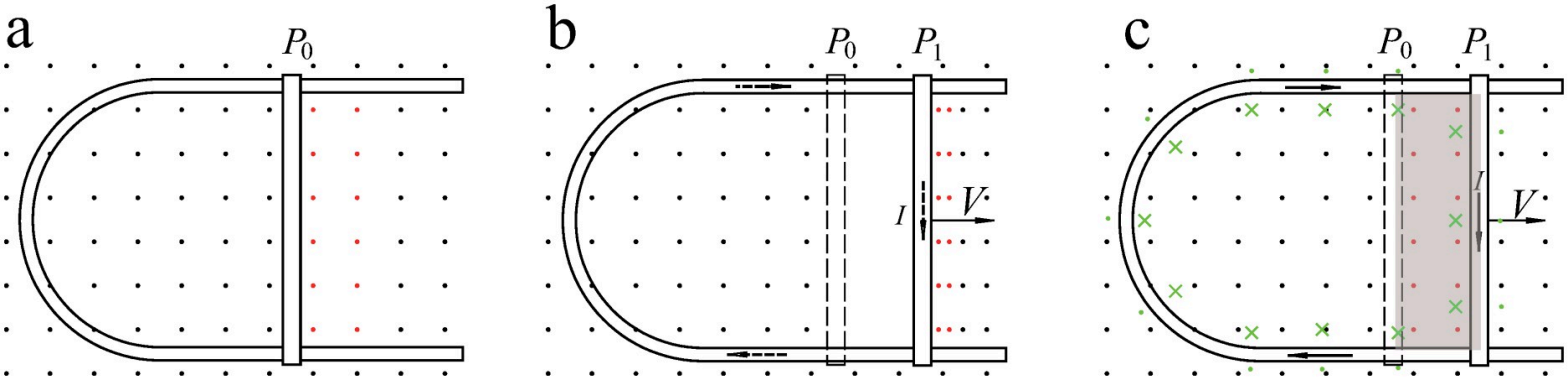
Generation of induced current. Both ends of the moving conducting rod are short-circuited by the fixed U-shaped conductor. (a) the initial position of the rod. (b) the field lines (in red) are assumed to be pushed to right. (c) the rod is assumed to cross the field lines (in red).

From Fig 11, the relationship between the induced current and the movement of the conductor is analyzed. To obtain a general relation, we first assume that the conductor of the system is not ideal. We then discuss the situation of an ideal conductor.

Before beginning our analysis, we distinguished the exogenic magnetic field from the endogenic magnetic field. The exogenic magnetic field is the magnetic field that existed before the induced current appeared (Fig 11a). The endogenous magnetic field (in green, Fig 11c) is generated by inducing an eddy current in the conductor.

In the loop,

$$\begin{array}{c} \frac{d\phi_{exogenic}}{dt}=\frac{d\phi_{endogenic}}{dt}+RI, \end{array}$$ (1)

where *ϕ*_*exogenic*_ denotes the exogenic magnetic flux inside the conductor loop and the term on the left-hand side is the motional electromotive force caused by the rod cutting the exogenic magnetic field. *ϕ*_*endogenic*_ denotes endogenic magnetic flux inside the loop. The first term on the right-hand side is the induced electromotive force (i.e., counter electromotive force) arising from the change in endogenic magnetic flux. The second term is the voltage drop arising from the current *I* through a resistor of resistance *R*.

In the simplest case, Eq 1 becomes

$$\begin{array}{c} BLV=\frac{d(\alpha I)}{dt}+RI, \end{array}$$ (2)

where *BLV* represents the motional electromotive force, *B* is the magnetic field strength, *L* is the length of the rod, *V* is the speed of the moving rod, and *α* is a coefficient equal to the endogenic magnetic flux generated by the unit current. This coefficient is related to the magnetic permeability of the system and area inside the loop. We assumed that the area swept by the rod was much smaller than the area inside the loop; therefore, *α* was assumed to be constant.

After rearranging Eq 2, we obtain an ordinary differential equation,

$$\begin{array}{c} \frac{dI}{dt}+\frac{R}{\alpha }I-\frac{BLV}{\alpha }=0. \end{array}$$ (3)

Assuming the induced current is initially zero, the solution is

$$\begin{array}{c} I=\frac{BLV}{R}(1-e^{-Rt/\alpha }). \end{array}$$ (4)

For convenience, we set all constants (*B*, *L*, *V* and *α*) equal to 1 in the plots; then,

$$\begin{array}{c} I=\frac{1-e^{-Rt}}{R}. \end{array}$$ (5)

**Fig 12:**
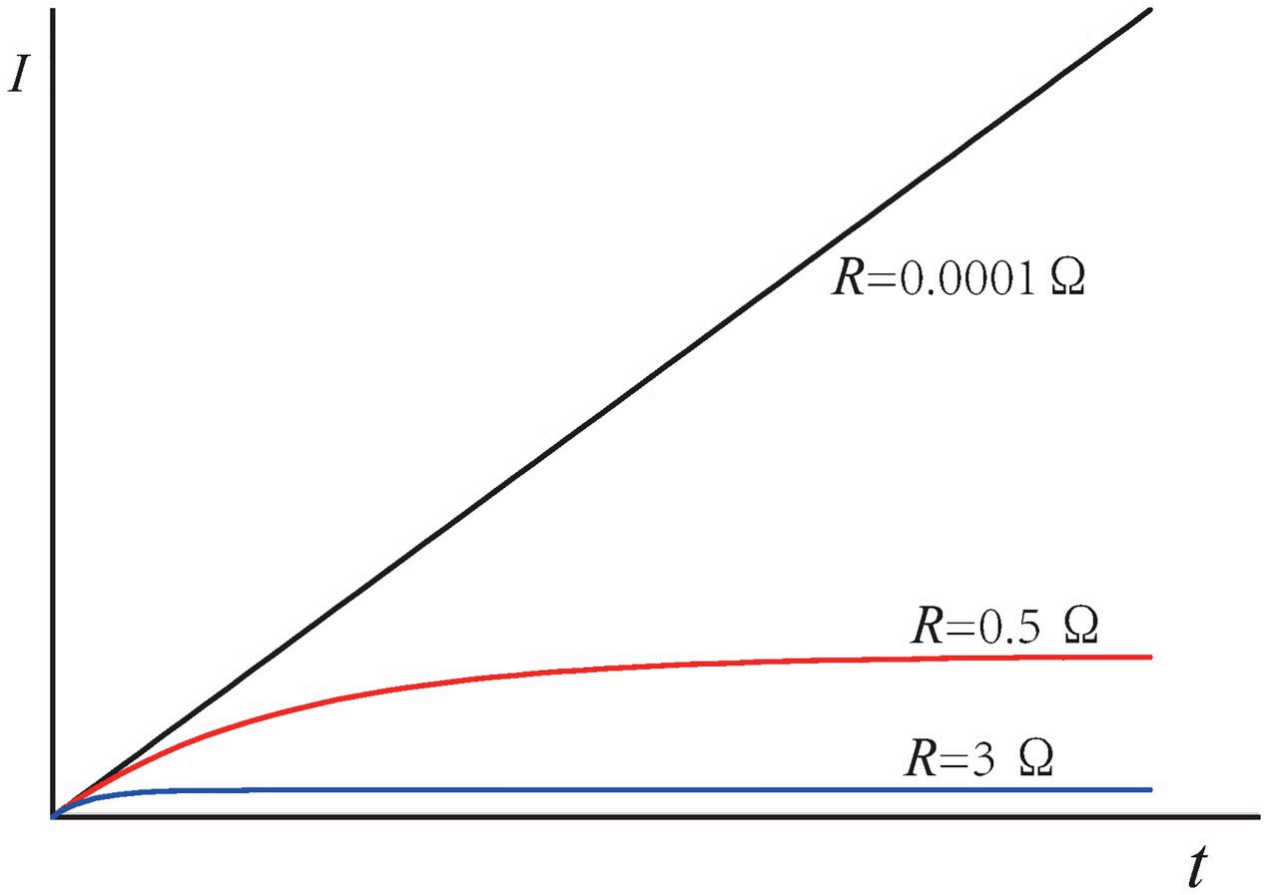
Relationship between the current and time.

**Fig 13:**
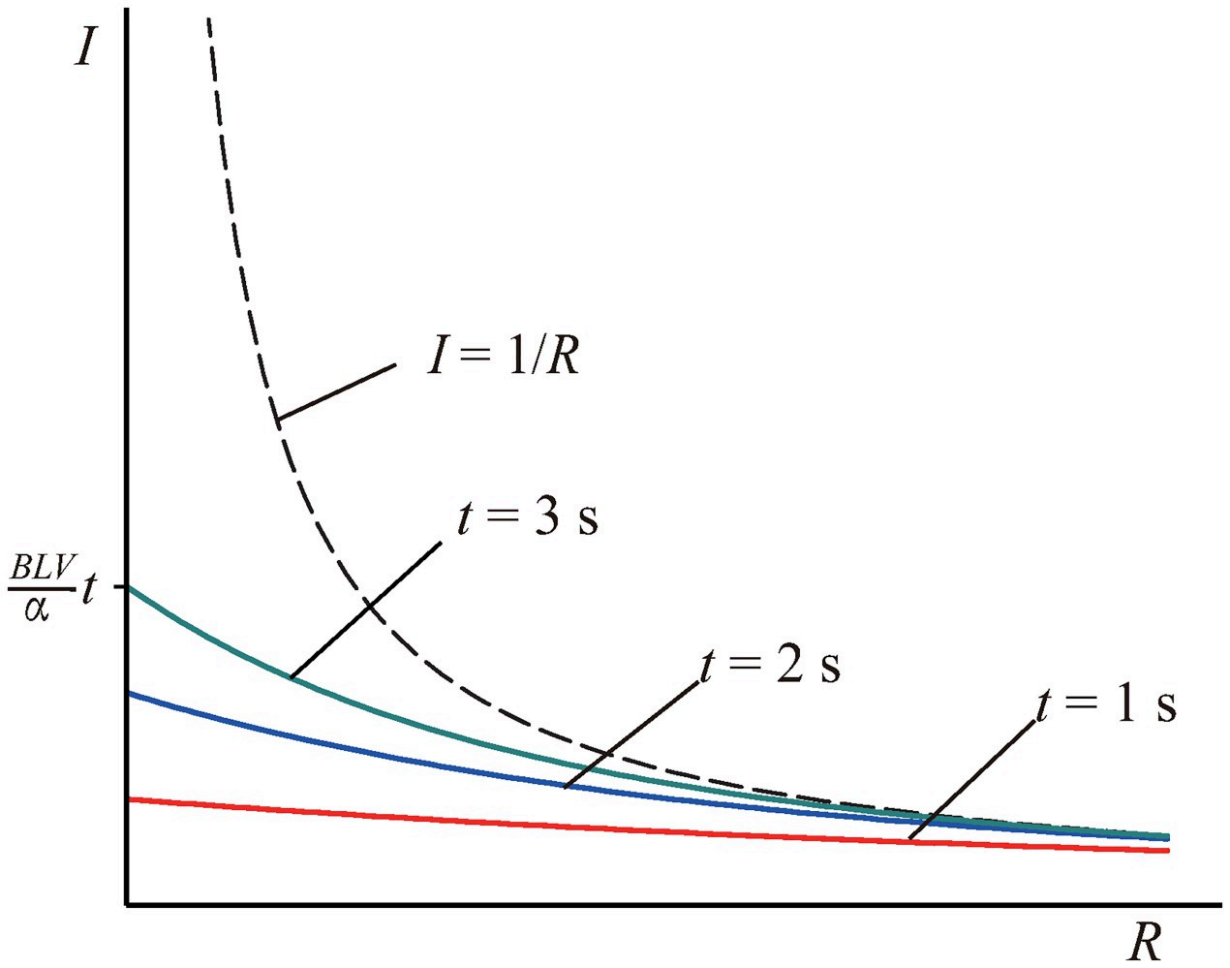
Relationship between the current and resistance. The frozen-in theorem advocates the dashed line; the relationship ascribed to in this study follows the solid lines.

Figs 12 and 13 are based on Eq 5. Fig 12, in the case of a large resistance, the current is negatively correlated with resistance. When the resistance tends to zero, the current tends to be independent of resistance, but is proportional to time *t*. Therefore, unless the elapsed time is infinite, the current is always limited.

$$\begin{array}{c} \underset{R\to 0}{\text{lim}}I=\underset{R\to 0}{\text{lim}}\frac{BLV}{R}(1-e^{-Rt/\alpha })=\frac{BLV}{\alpha }t. \end{array}$$ (6)

The meaning of this limit is illustrated in Fig 13. *BLVt* is the exogenic magnetic flux in the shaded area, as shown in Fig 11c. Therefore, for an ideal conductor, the maximum current is related to the exogenic magnetic flux swept by the rod. Alternatively, the current is related to the rod position. The farther the rod moves, the greater is the current. When the rod stops moving, the current remains constant because there is no resistance and no energy consumption. This also indicates that if the rod does not move indefinitely, the induced current is always finite.

If the resistance is high, the **speed** of the rod determines the current; however, if the resistance is zero, the **position** of the rod determines the current.

By using expertise of electronic engineering, we can obtain a better understanding of the results. If the resistance is large, the current is determined by the resistance. If the resistance is small, the current is determined by the inductance. If the resistance is negligible, then the inductance and capacitance dominate. The role of the capacitance was discussed in LL2015.

The reason given by Alfvén [1] when he proposed the frozen-in theorem was

“*In view of the infinite conductivity, every motion (perpendicular to the field) of the liquid in relation to the lines of force is forbidden because it would give infinite eddy currents. Thus the matter of the liquid is ‘fastened’ to the lines of force*….”

To avoid an infinite current, Alfvén forbids the ideal conductive fluid from crossing the magnetic field lines and allows the fluid to freeze together with the magnetic field lines. Obviously, he overlooked the important role of the inductance. This is the source of error in the frozen-in theorem.

When proposing the frozen-in theorem, Alfvén did not distinguish between the endogenic and exogenic magnetic fields. Now, we must realize that in an ideal conductor, only the sum of the endogenic and exogenic magnetic fluxes is conserved. The exogenic and endogenic magnetic fluxes are both variable and complementary. This complementary relationship can be seen from Eq 1: If *R* = 0, the change rates of the endogenic and exogenic magnetic fluxes are exactly equal.

**Fig 14:**
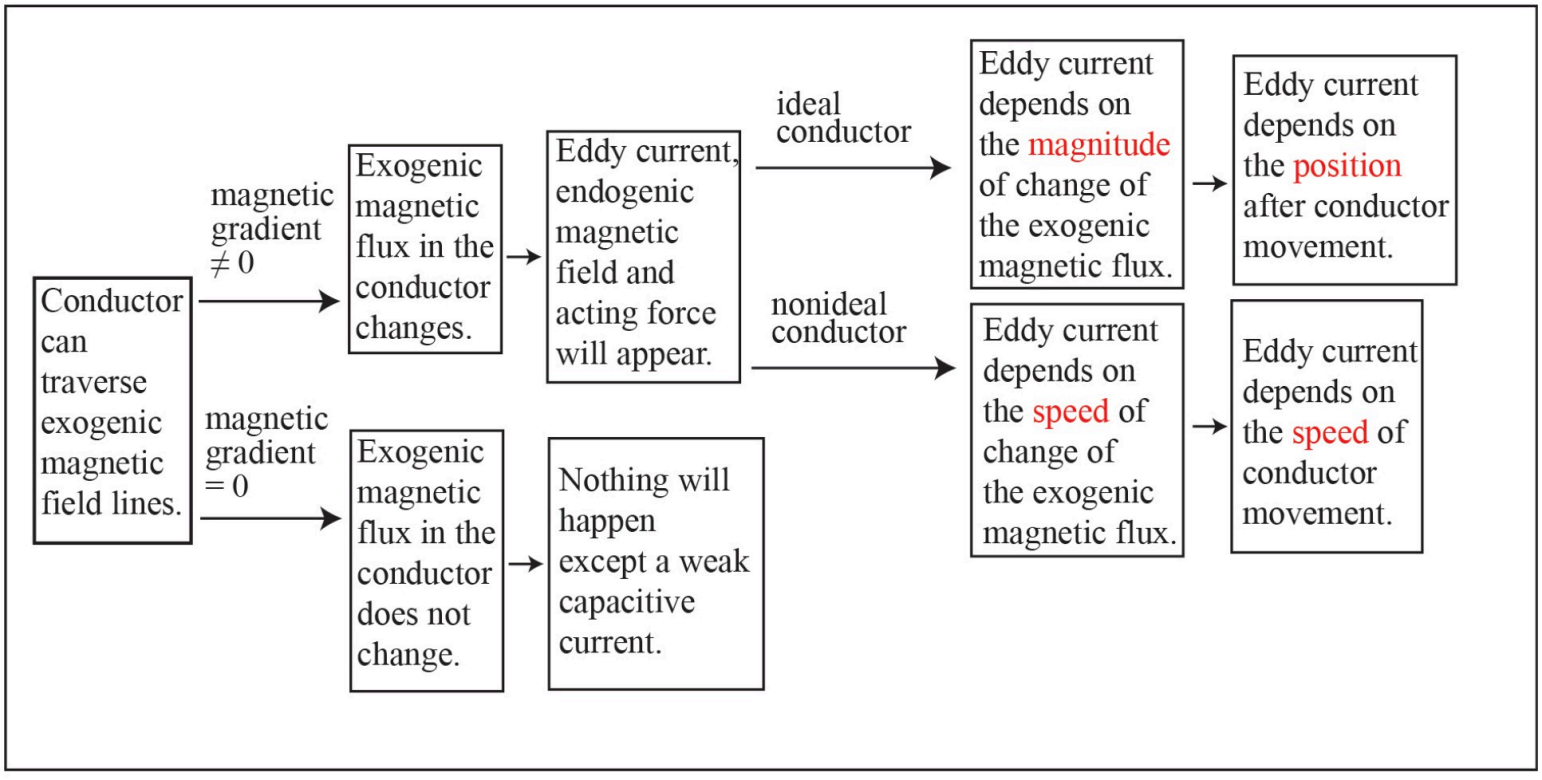
Thought diagram.

The above analysis results can be summarized in Fig 14.

## Conclusion

Our experiments have proved two points:

1. When a conductor moves perpendicular to the magnetic field lines, the front magnetic field lines may become sparser (Fig 7) and the rear magnetic field lines may become denser (Fig 6). This result contradicts the frozen-in theorem.
2. When a conductor crosses a uniform magnetic field, the conductor does not disturb the magnetic field, and the magnetic field does not impose magnetic resistance on the conductor. This result also contradicts the frozen-in theorem.

Therefore, these results prove that the frozen-in theorem is wrong.

Our analysis shows that, if inductance is also considered, the motional electromotive force generated by the ideal conductor (or fluid) crossing the magnetic field lines will not cause an infinite current. Therefore, the frozen-in theorem is based on incorrect foundations.

In conclusion, the frozen-in theorem should be abandoned.
